# The role of the *Cronobacter sakazakii* ProP C-terminal coiled coil domain in osmotolerance

**DOI:** 10.1186/s13099-014-0046-9

**Published:** 2014-12-16

**Authors:** Audrey Feeney, Christopher D Johnston, Alan Lucid, Jim O’Mahony, Aidan Coffey, Brigid Lucey, Roy D Sleator

**Affiliations:** Department of Biological Sciences, Cork Institute of Technology, Rossa Avenue, Bishopstown, Cork Ireland

## Abstract

**Background:**

We investigate the role of the C-terminal coiled coil of the secondary proline porter ProP in contributing to *Cronobacter sakazakii* osmotolerance.

**Findings:**

The extended C-terminal domain of ProP1 (encoded by ESA_02131) was spliced onto the truncated C-terminal end of ProP2 (encoded by ESA_01706); creating a chimeric protein (ProPc) which exhibits increased osmotolerance relative to the wild type.

**Conclusions:**

It appears that the C-terminal coiled coil domain tunes ProP at low osmolality, whereas ProP transporters lacking the coiled coil domain are more active at a higher osmolality range.

## Introduction

Survival of the foodbourne pathogen *Cronobacter sakazakii* in low water activity (a_w_) environments, e.g. powdered infant formula (PIF), is largely attributed to the accumulation of organic compounds termed osmolytes or compatible solutes [1,2]. Synthesised *de novo*, or transported from the bathing solution[3], compatible solutes function to increase cell turgor thereby counterbalancing the external osmotic upshift and preventing water loss from the cell, which if left unchecked can lead to plasmolysis and ultimately cell death [4].

In *Escherichia coli*, a model organism for the study of bacterial osmoadaptation, the transmembrane protein ProP is perhaps the best characterised compatible solute uptake system; facilitating the uptake of both proline and glycine betaine [5]. A member of the major facilitator superfamily (MFS), *E. coli* ProP is a 500-amino acid protein comprising of 12 transmembrane domains and a characteristic carboxy-terminal extension [5,6]. In a previous *in silico* study we identified seven ProP homologues on the *C. sakazakii* BAA-894 genome; one of which, ESA_02131, encodes a protein exhibiting 90% identity to *E. coli* ProP [2]. While the remaining six homologues encode proteins exhibiting features of classic secondary transporters, they are all 60–70 amino acids shorter than the *E. coli* ProP; lacking the extended carboxyl tail [2]. Notwithstanding the lack of structural consistency, particularly at the C-terminal end, we have shown that six of the seven *C. sakazakii proP* homologues contribute to *C. sakazakii* osmotolerance, albeit to varying degrees [7].

Culham et al. [5] first described the *E. coli* ProP as harbouring unusual structural features which appeared unique within the transporter superfamily. This study predicted the formation of an alpha helical coiled coil resulting from the presence of the carboxyl terminal extension [5]. Indeed, a synthetic polypeptide corresponding to the C-terminal extension of ProP formed a dimeric alpha helical coiled coil [6]. Interestingly, when amino acid changes were introduced to the coiled coil, ProP required a larger osmotic upshift to become activated [6], suggesting that the C-terminal domain likely plays a role in osmosensing. Furthermore, a derivative of ProP which lacked the 26 amino acid C-terminal domain was expressed, but inactive [6]. In contrast, despite the structural degeneracy observed between the homologues, *C. sakazakii* ProP homologues lacking the C-terminal extension do contribute to osmotolerance, albeit to a lesser extent than the extended ProP (which we designate Prop1) encoded by ESA_02131 [7].

While several studies have focused on elucidating the role of the carboxyl extension in *E. coli* [5,6,8], little is known about the role, if any, of the ProP1 carboxyl extension in the far more osmotolerant *C. sakzakii*. Herein, we investigate the role of the C-terminal coiled coil of ProP1 in contributing to *C. sakazakii* osmotolerance, by creating a chimeric protein (ProPc) in which the extended C-terminal domain of ProP1 (encoded by ESA_02131) is spliced onto the truncated C-terminal end of ProP2 (encoded by ESA_01706).

## Material and methods

### Bacterial strains and growth conditions

Bacterial strains and plasmids used in this study are listed in Table 1.

**Table 1:** **Bacterial strains and plasmids**

***Strain or plasmid***	***Relevant genotype or characteristics***	***Source or reference***
**Plasmids**		
pUC18	Amp^r^, lacZ', pMB9 replicon	[9]
pUC18: ESA_02131	pUC18 harboring ESA_02131 gene under control of the native promoter	[7]
pUC18: ESA_01706	pUC18 harboring ESA_01706 gene under control the native promoter	[7]
pUC18: ESA_01706CTE	pUC18 harboring chimeric ESA_01706 with fused C-terminal extension (ESA_02131) under control of the native promoter	This work
**Strains**		
*Cronobacter sakazakii* BAA-894	*C.sakazakii* strain isolated from powdered formula associated with neonatal intensive care unit	[10]
*Escherichia coli* DH5α	Intermediate cloning host.supE44 ΔlacU169(80lacZΔM15)R17 recA1 endA1 gyrA96 thi-1 relA1	Invitrogen
MKH13	MC4100Δ(putPA)101Δ(proP)2Δ(proU)	[11]
MKH13 pUC18:ESA_02131+	Host strain harbouring pUC18: ESA_02131 plasmid. Amp^r^	[7]
MKH13 pUC18:ESA_01706+	Host strain harbouring pUC18: ESA_01706 plasmid. Amp^r^	[7]
MKH13 pUC18:ESA_01706CTE	Host strain harbouring pUC18: ESA_01706CTE plasmid. Amp^r^	This work

Amp^r^. This strain is resistant to ampicillian.

### Creation of the chimeric ProPc protein

PCR primers (Table 2) were designed for each *proP* homologue based on *C. sakazakii* strain BAA-894 sequence data available from the NCBI database (NC_009778.1). The formation of the chimeric ProP protein (ProPc), which consists of the extended coiled coil region of ProP1 (amino acid position 422 to 505) fused to the C-terminus of ProP2 (encoded by ESA_01706), was performed using a modified SOEing (Splicing by overlap extension) technique [12]. *In silico* comparative analysis of the native ProP1 and ProP2 sequences, revealed a point of amino acid homology within the twelfth predicted transmembrane domain, a leucine/isoleucine/threonine triplet (LIT) at position 422–424 and 437–439 respectively, which was selected as the splice site. Briefly, the fusion was performed using three separate PCR reactions: the first PCR (primer set Chimeric-01706) resulted in an ESA_01706 (*proP2*) amplicon lacking the C-terminal extension but containing a 15-bp 3’overhang corresponding to the LIT triplet of the ProP1 C- terminal extension. The second PCR (primer set Chimeric-02131CTE) formed an amplicon of 210-bp encoding ProP1 C-terminal extension with a 5’-overhang, also corresponding to the LIT triplet. The final PCR (primer set Chimeric-01706-F Chimeric-02131tail-R) was performed with the two previous amplicons as template; resulting in a final product of 1,623-bp, representing the ESA_01706 native promoter and modified coding region (encoding the fused ProP1 C-terminal extension after the LIT triplet). This product was digested with restriction enzymes BamHI and HindIII and ligated to a similarly digested pUC18 vector forming pUC18:ESA_01706CTE (**C**-**T**ermini **E**xtension). The integrity of the chimeric sequence was confirmed by sequencing (MWG Operon, Germany and GATC, Germany) and transformed into *E. coli* MKH13.

**Table 2:** **Primers**

***Primer name***		***Primer sequence (5' to 3')***	***Length***	***Characteristics***
ESA_02131	F	CATCGGCCGACAGGCCAGTCAATGAATGATGC	32	EagI cut site
	R	CATTCTAGAGAGTACAACGGAATGCGGGG	29	XbaI cut site
ESA_01706	F	CATTCTAGAGTCGGGCGGCTCTTTATCTGG	30	XbaI cut site
	R	CATGGATCCTTGACCAGATGACGCAGTCTTTC	32	BamHI cut site
Chimeric-01706	F	AATAAGCTTGTGGCTTTTTATGCCGGGCTGC	31	HindIII cut site
	R	CAGGCCAGTAATCAGCGCCGCGCCCATGAC	30	3' SOEing overhang
Chimeric-02131CTE	F	CGCGGCGCTGATTACTGGCCTGACGATGAAAG	32	5' SOEing overhang
	R	AATGGATCCTTACTCGTTAATACGAGGATGCTGG	34	BamHI cut site
pUC18 MCS Check	F	CATTAGCTCACTCATTAGGCACC	23	pUC18 insert check
	R	CATTGTAAAACGACGGCCAGTG	22	pUC18 insert check

### Osmotolerance assay

Overnight cultures of *E. coli* MKH13 clones expressing the wild-type and chimeric ProP proteins (ProP1, ProP2 and ProPC respectively) were grown at 37°C with shaking at 200 rpm in either 10 ml LB or M9 minimal media containing 0.5% glucose, 0.04% arginine, 0.04% isoleucine, 0.04% valine (Sigma-Aldrich Co.). Cells were pelleted by centrifugation at 5,000 g, washed and re-suspended in 200 ***μ***l ¼ strength Ringer’s solution. The cell suspension was added to the appropriate filter sterilized media with varying concentrations (0-10%) of added NaCl. Growth was monitored in the relevant media over a 48 hour period, with optical density (OD_600_) readings being taken every hour. Triplicate readings were taken and graphs were plotted using SigmaPlot version 11.0. *E. coli* MKH13 harbouring the empty pUC18 plasmid was used as a negative control.

## Results

### *C. sakazakii* ProP structures

**Figure 1:**
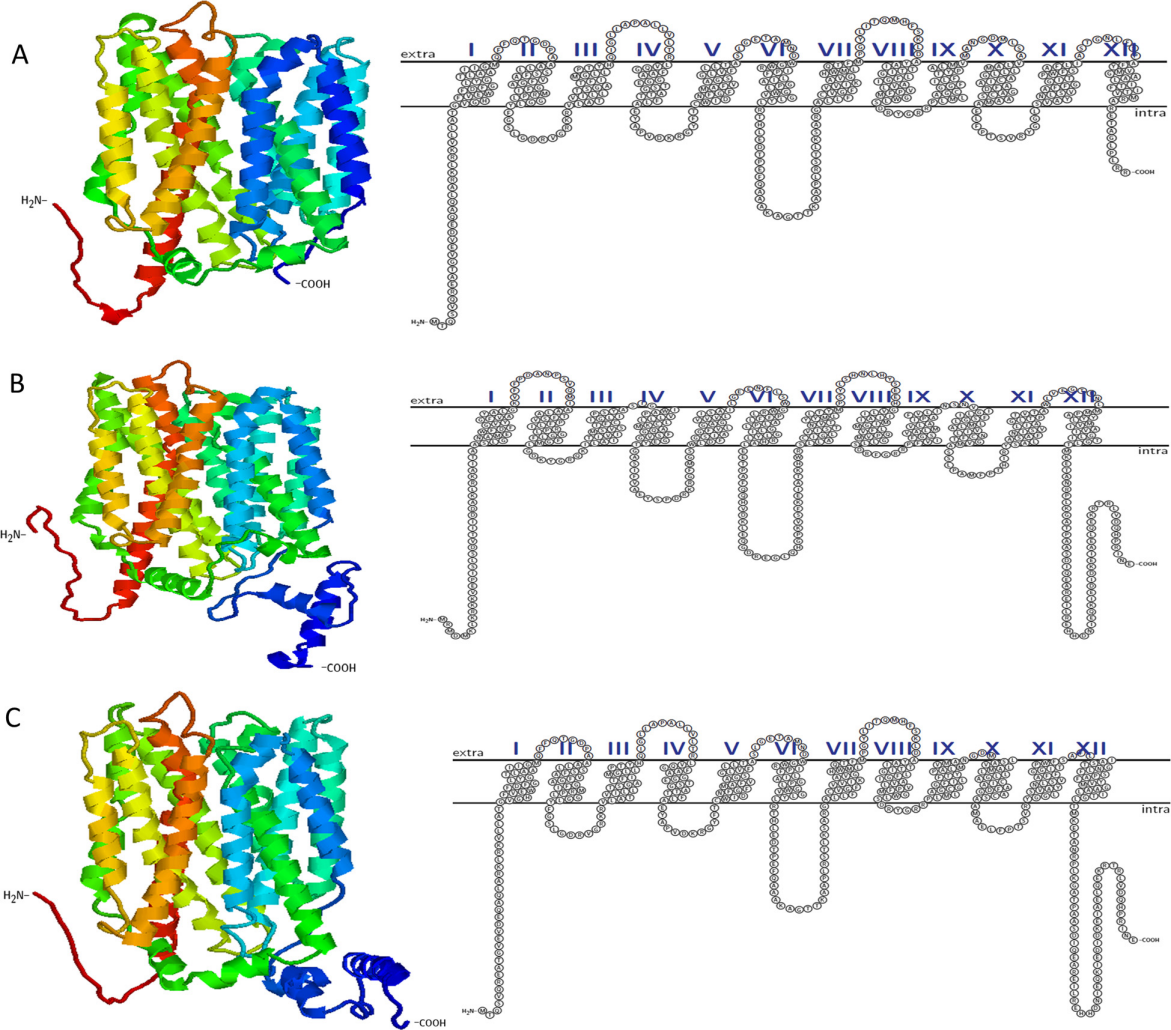
**Predicted transmembrane and tertiary structures of A) ProP2 encoded by ESA_01706, B) ProP1 encoded by ESA_02131 and C) ProPc.**

Based on sequence similarity to the *E. coli* ProP protein, we identified ProP1 (the product of ESA_02131) as the most likely ProP homolog in *C. sakazakii*; exhibiting 90% amino acid sequence identity and structural features characteristic of *E. coli* ProP. Indeed, further analysis using TMHMM and TexTopo software predicted ProP1 to be a membrane protein with 12-transmembrane domains, an extended central hydrophilic loop and carboxy terminal extension (Figure 1). While the remaining five ProP homologues on the *C. sakazakii* BAA-894 genome were also predicted to encode proteins with 12 transmembrane domains and an extended central hydrophilic loop, they each lacked the extended carboxy-terminal domain identified in ProP1, a feature which likely affects the final protein structure and function.

Figure 1B illustrates the tertiary structure for ProP1 (predicted using the I-TASSER server [13,14]). Most notably the presence of a coiled coil domain is evident as a result of the extended carboxy-terminal identified by sequence analysis. The coiled coil domain likely protrudes into the intracellular cytoplasm of the organism where its function remains unclear. By contrast, the tertiary structure of ProP2 (Figure 1A), representative of the remaining 6 ProP homologues and exhibiting 40% identity to *E. coli* ProP and 49% identity to ProP1, lacks the coiled coil domain at the carboxy-terminal end.

### Chimeric protein (ProPc) expression in *E. coli* MKH13

The osmoprotective properties of ProP1, ProP2 and ProPc were measured and compared in *E. coli* MKH13; an osmosensitive mutant incapable of growth in high osmolality environments (≥4%). The pUC18 plasmid containing each gene of interest was transformed to *E. coli* MKH13. Transformation efficiencies of 60 CFU/μg DNA were achieved, with successful transformation being confirmed by colony PCR, followed by sequencing. Transformants were screened for osmotolerance on media (both LB and M9 plus 1 mM proline) containing between 4% and 10% added NaCl.

### Assessment of osmotolerance

To determine the effect of each ProP homologues, both native (ProP1 and ProP2) and chimeric (ProPc), on the osmotolerance of *E. coli* MKH13, each of the strains was grown in media containing varying concentrations of NaCl. Growth was monitored over a 48 hour period in minimal media supplemented with 1 mM proline and containing 0-10% added NaCl.

**Figure 2:**
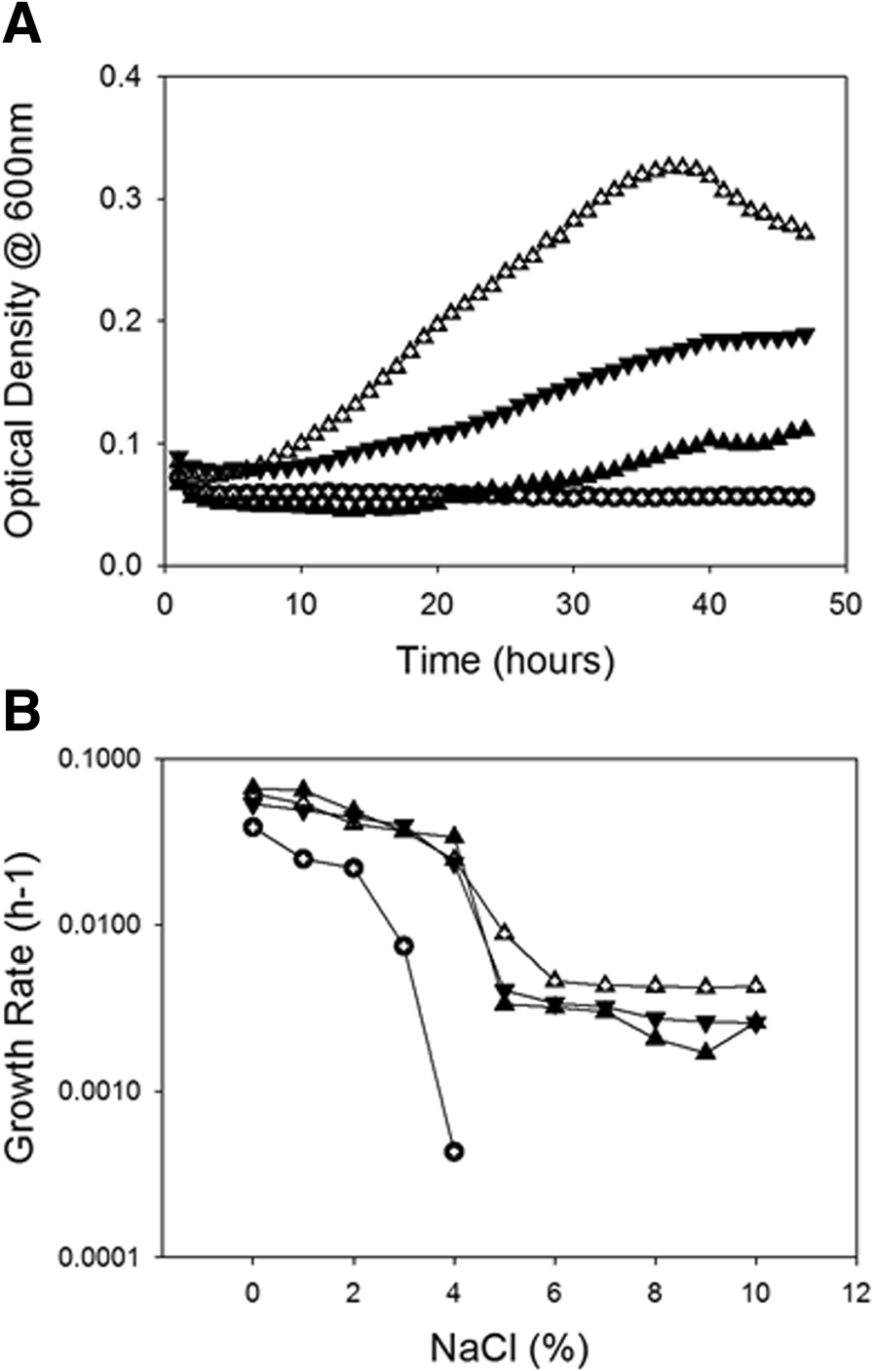
**Physiological analysis of** ***E. coli*** **MKH13 expressing**  **ProP1,**  **ProP2,**  **ProPc and**  **the empty pUC18 plasmid. A)** Optical density was measured over a 48 hour period in media supplemented with 0-10% added NaCl. **B)** The growth rate of *E. coli* MKH13 expressing each of the proteins of interest was measured in media supplemented with 0-10% added NaCl.

Media containing 5% NaCl yielded the most discriminatory results. While *E. coli* MKH13 expressing the empty pUC18 vector showed no growth at 5%, each of the other three strains tested conferred some degree of osmotolerance on the host (Figure 2A). The strain expressing ProP1 was the most osmotolerant, with a maximum optical density (OD_600_) of 0.326 after 37 hours growth at 5% NaCl. The strain possessing ProPc grew to an OD significantly higher than the strains expressing either ProP1 or ProP2. *E. coli* MKH13 expressing ProP2 in 5% NaCl grew to a maximum OD of 0.111 after 48 hours, whereas *E. coli* MKH13 containing ProPc grew to a final OD of 0.189 at the same time point. Interestingly, growth of the stains expressing ProP2 and the chimeric protein continued to increase up to 48 hours, while growth of *E. coli* MKH13 expressing ProP1 reached maximum OD after only 37 hours.

Each *E. coli* MKH13 strain expressing a *proP* gene of interest conferred osmotolerance (Figure 2B). As expected, *E. coli* MKH13 demonstrated a significant reduction in growth rate as NaCl concentrations increased, with a final growth rate of 0.0004 hr^−1^ recorded in media supplemented with 4% NaCl and no subsequent growth recorded thereafter. *E. coli* MKH13 expressing ProP1 demonstrated the highest osmotolerance of all the strains tested with growth rates of 0.009 hr^−1^ to 0.004 hr^−1^ recorded in media supplemented with 5% to 10% NaCl respectively. The next most osmotolerant strain was that expressing ProPc. Growth rates of 0.004 hr^−1^ to 0.003 hr^−1^ were recorded in media supplemented with 5% to 10% NaCl respectively (Table 3). This was higher than the growth rates observed when *E. coli* MKH13 expressing the native ESA_01706 gene (ProP2) was grown in a high osmolality environment, suggesting an important role for the *C. sakazakii* ProP C-terminal extension in osmotolerance.

**Table 3:** **Growth rate and optical density @ 600 nm**

***Protein expressed***	***Gene locus tags***	***Name***	***5%***		***6%***		***7%***		***8%***		***9%***		***10%***	
			***Max. OD***	***Growth rate (hr*** ^***−1***^ ***)***	***Max. OD***	***Growth rate (hr*** ^***−1***^ ***)***	***Max. OD***	***Growth rate (hr*** ^***−1***^ ***)***	***Max. OD***	***Growth rate (hr*** ^***−1***^ ***)***	***Max. OD***	***Growth rate (hr*** ^***−1***^ ***)***	***Max. OD***	***Growth rate (hr*** ^***−1***^ ***)***
Native	ESA_02131	ProP1	0.326	0.009	0.144	0.005	0.152	0.004	0.177	0.004	0.181	0.004	0.204	0.004
Native	ESA_01706	ProP2	0.111	0.003	0.096	0.002	0.083	0.002	0.074	0.002	0.127	0.002	0.171	0.002
Chimeric	ESA_02131 ESA_01706	ProPc	0.189	0.004	0.130	0.003	0.113	0.003	0.117	0.003	0.124	0.003	0.141	0.003

## Discussion

A unique feature of the neonatal pathogen *C. sakazakii* is its ability to survive for prolonged periods in environments of low a_W_, such as powdered infant formula (PIF), making it a significant cause for concern [15]. Indeed, up to 80% of infants infected with *C. sakazakii* die within days of birth, while survivors often suffer delayed neurological symptoms, brain abscesses or hydrocephalus [16,17]. However, despite this, little is known about the molecular mechanisms that allow this organism to survive in environments such as PIF where it is subject to extreme hyper-osmotic stress.

In a previous *in silico* study we identified seven copies of an *E. coli proP* homolog on the BAA-894 genome. Physiological analysis confirmed that six of the *proP* homologues identified played a role in osmotolerance. The availability of osmolytes in the media also had an effect on the osmotolerance of the host, with growth rates varying depending on the type or variety of compatible solutes present [7]. While all six ProP proteins exhibited features characteristic of classic secondary transporters, five of the proteins were between 60–70 amino acids shorter than ProP1; lacking the characteristic C-terminal cytoplasmic extension Previous studies in our lab have demonstrated that the six *C. sakazakii* ProP homologues lacking the C-terminal coiled coil are significantly less osmoprotective than ProP1 [7], suggesting an important role for this domain in modulating *C. sakazakii* osmotolerance.

In the current study, the *C. sakazakii* ProP2 (encoded by ESA_01706) was chosen as the prototypical ProP homologue to study the role of the alpha helical coiled coil in osmotolerance. Genetic splicing yielded a chimeric protein structure (ProPc) possessing the native ProP2 domains in addition to the C-terminal alpha helical coiled coil domain from ProP1 (Figure 1C). *E. coli* MKH13 expressing ProPc grew to a higher OD in minimal media supplemented with proline, when compared to the native protein ProP2 which lacked the extended coiled coil domain (Figure 2A). These data demonstrate that the addition of the coiled coil domain from ProP1 to ProP2 results in a protein with an increased osmoprotective effect on the usually osmotically sensitive *E. coli* MKH13. However, as the osmolality of the medium increased, this trend appeared to reverse with OD readings becoming similar at 9% NaCl and the chimeric protein growing to a higher OD than the native in 10% NaCl, suggesting that the extent of osmotic pressure also has a role to play in the activity of the proteins.

The role of the C-terminal domain of other osmolyte transporters, such as BetP (*Corynebacterium glutamicum*) and OpuA (*Bacillus subtilis*), was demonstrated to be important for the activation of these proteins during an increase in the osmolality of the surrounding medium [18]. Furthermore, Culham et al. created a synthetic polypeptide corresponding to the C-terminal domain of *E. coli* ProP which formed a dimeric alpha helical coiled coil structure [6], similar to the coiled coil structure of ProP1 (illustrated in Figure 1A). In the same study ProP proteins from both *E. coli* and *Agrobacterium tumefaciens,* possessing the characteristic alpha helical coiled coil, were activated at a lower osmolality than orthologues lacking the coiled coil structure. *C. glutamicum* possesses a ProP protein which lacks the C-terminal alpha helical coiled coil domain and, presumably as a result of this, requires a higher osmolality for activation [6]. *E. coli* ProP variants lacking the coiled coil or with an amino acid substitution disrupting the formation of the alpha helical coiled coil, also require a larger osmotic upshift than the wild-type transporter [6,19]. This study demonstrates that the activity of these ProP orthologues is dependent on the osmolality of the surrounding medium, and the alpha helical coiled coil is believed to tune the transporter to osmoregulate the cell over a low osmolality range [19]. These data may therefore offer an explanation for the increased growth observed in *E. coli* MKH13 expressing ProP2, which lacks the coiled coil domain, in media supplemented with 10% NaCl relative to either ProP1 or ProPc (Figure 2). It is likely that the coiled coil domain of *C. sakazakii* ProP1 has a similar tuning function. Furthermore, the presence of multiple ProP porters lacking the C-terminal coiled coil domain, and therefore only active at a higher osmolality, may well explain the extreme osmotolerance unique to *C. sakazakii*; allowing the pathogen to survive in environments like PIF. The ProP1 protein, on the other hand possessing the coiled coil, may be the only osmolyte transporter required to respond to low or moderate hyperosmotic challenge.

## Conclusion

The addition of the coiled coil domain from ProP1 to ProP2 resulted in a chimeric protein (ProPc) which demonstrated higher osmotolerance compared to the native ProP2 (under moderate osmotic stress conditions). Furthermore, the growth rate of *E. coli* MKH13 expressing ProP2 increased in minimal media supplemented with 10% NaCl; suggesting that, as is the case in *E. coli* [19], the coiled coil domain tunes ProP at low osmolality, whereas ProP transporters lacking the coiled coil domain are more active at a higher osmolality range.
